# Health Insurance and Its Psychosocial Correlates in Patients with Advanced Lung Cancer in Japan

**DOI:** 10.31662/jmaj.2024-0289

**Published:** 2025-06-13

**Authors:** Fumimaro Ito, Takashi Sato, Kohei Horiuchi, Daisuke Arai, Keiko Ohgino, Kota Ishioka, Hideki Terai, Shinnosuke Ikemura, Hiroyuki Yasuda, Ichiro Nakachi, Ichiro Kawada, Takashi Inoue, Yoshitaka Oyamada, Takeshi Terashima, Koichi Sayama, Daisuke Fujisawa, Mari Takeuchi, Koichi Fukunaga, Katsuhiko Naoki, Kenzo Soejima

**Affiliations:** 1 Department of Medicine, Keio University School of Medicine, Tokyo, Japan; 2 Department of Respiratory Medicine, Kitasato University School of Medicine, Sagamihara, Japan; 3 Pulmonary Division, Internal Medicine, Saiseikai Utsunomiya Hospital, Utsunomiya, Japan; 4 Department of Internal Medicine, Tokyo Saiseikai Central Hospital, Tokyo, Japan; 5 Department of Pulmonary Medicine, Faculty of Medicine, University of Yamanashi, Yamanashi, Japan; 6 Department of Internal Medicine, Division of Pulmonary Medicine, Iwate Medical University School of Medicine Morioka, Japan; 7 Department of Internal Medicine, Sano-Kosei General Hospital, Sano, Japan; 8 Department of Respiratory Medicine, National Hospital Organization Tokyo Medical Center, Tokyo, Japan; 9 Department of Respiratory Medicine, Tokyo Dental College Ichikawa General Hospital, Ichikawa, Japan; 10 Division of Pulmonary Medicine, Kawasaki Municipal Kawasaki Hospital, Kawasaki, Japan; 11 Department of Neuropsychiatry, Keio University School of Medicine, Tokyo, Japan; 12 Division of Patient Safety, Keio University School of Medicine, Tokyo, Japan; 13 Palliative Care Center, Keio University Hospital, Tokyo, Japan

**Keywords:** health insurance, private health insurance, lung cancer, quality of life, Japan

## Abstract

**Introduction:**

Japan has a national health insurance system that covers at least 70% of regular medical costs and provides additional benefits for high medical costs. In addition, >60% of the population holds private health insurance to reduce financial toxicity. However, there has been a lack of real-world data on private health insurance in the oncology setting in Japan.

**Methods:**

A cross-sectional survey of health insurance was conducted at 16 hospitals in Japan between 2013 and 2016. Patients were eligible if they were newly diagnosed with clinical stage IIIB or IV lung cancer. Data collected included patients’ health insurance, clinical and sociodemographic characteristics, and self-reported outcomes 3 months after diagnosis.

**Results:**

Of the 147 patients, 114 (77.6%) had private health insurance. Patients with private health insurance were significantly younger (p = 0.028), had better performance status (p = 0.029), and reported better quality of life (p = 0.017), specifically in social (p = 0.039) and emotional (p = 0.011) well-being. There were no significant associations between having private health insurance and treatment details, medical satisfaction, or financial issues. Most patients (99.3%) reported that national health insurance is necessary. A substantial proportion of patients (9.8%), particularly those without private health insurance (19.4%), reported that private health insurance is not necessary.

**Conclusions:**

Having private health insurance was associated with better quality of life, though there were no significant differences in medical care or financial issues. Our findings suggest that private health insurance plays an auxiliary role in accessing medical care for patients with advanced lung cancer in Japan.

## Introduction

Patients with advanced cancer experience a variety of psychosocial issues, which are associated with their quality of life (QOL) ^(1), (2)^. Among these, financial burdens are especially severe early in cancer treatment and are also linked to worse health-related QOL in patients ^(3), (4)^. In an era when medical advances have improved treatment outcomes, including survival rates for various cancers, financial toxicity has emerged as a global threat. Having health insurance is considered the most effective way to reduce financial toxicity. For example, it has been reported that having supplemental insurance lowers out-of-pocket spending for cancer patients in the United States ^(5)^. However, the structure of national and private health insurance varies from country to country, and it is not clear how private health insurance assists patients in each country.

Japan achieved universal national health coverage in 1961, earlier than most other countries, and established a system in which almost all citizens receive the same benefits and pay the same percentages of medical costs ^(6)^. In the Japanese national health insurance system, the 6-69 age group receives 70% coverage, the 70-74 age group receives 80% coverage, the over-75 age group receives 90% coverage, and welfare recipients receive 100% coverage. Japan also has a high-cost medical care benefit system, in which medical costs exceeding a certain amount are reimbursed if out-of-pocket expenses for medical care become too high. These systems allow Japanese patients to receive medical care with minimal out-of-pocket expense. Although such systems are expected to incur significant costs, the ratio of government expenditure devoted to health as a percentage of gross domestic product was 11.1% in 2019, which is lower compared to other countries ^(7), (8)^. However, Japan faces a unique issue related to social insurance: the aging population has made it impossible to cover medical expenses solely through premiums from the working-age population ^(6)^. This problem is expected to grow as the population continues to age.

Due to the public health insurance system, private health insurance in Japan differs somewhat from that in other countries. In other countries, private health insurance typically covers services not included in statutory health care systems, improves access to services, and reduces patient cost-sharing requirements ^(9)^. In contrast, in Japan, although approximately 65% of the population holds private health insurance, it is primarily supplementary or complementary: it provides an insured person who is ill or hospitalized with additional income in the event of illness, either as a lump-sum or daily payments over a defined period ^(10), (11)^. General private insurance in Japan typically covers inpatient services for any disease, including cancer, but provides daily payments over a limited hospitalization period and does not cover outpatient treatment unless special contracts are added. On the other hand, cancer-specific insurance and cancer-specific contracts added to general insurance, which are now popular in Japan with a subscription rate of 39.1%, typically provide lump-sum benefits upon cancer diagnosis and medical care specifically for cancer.

However, little is known about how these private insurance policies benefit patients with advanced cancer in Japan. Despite this lack of knowledge, Japanese people tend to join private health insurance due to the influence of health insurance advertisements. Even the characteristics of patients with advanced cancer who have private health insurance remain unclear. Therefore, in this multicenter cross-sectional study, we aimed to investigate the reality of private insurance for patients diagnosed with advanced lung cancer in Japan and examined (1) the factors associated with having private insurance and (2) the association of having private insurance with self-reported QOL.

## Materials and Methods

### Study procedures and participants

This study is part of a larger investigation in which various psychosocial aspects of patients with advanced lung cancer were assessed. Details of the study procedure have been described elsewhere ^(2), (12), (13), (14)^. Patients were recruited from 16 academic or medium/large hospitals in Japan between December 2013 and March 2016. Patients were eligible if they were newly diagnosed with clinical stage IIIB or IV lung cancer (defined by the seventh edition of lung cancer stage classification ^(15)^), were ≥20 years of age, and could write and comprehend Japanese. Patients with significant cognitive impairment or who had already received any anticancer treatments, including chemotherapy, radiation, surgery, or immunotherapy, were excluded. This study was approved by the institutional review board of all participating hospitals (Keio University School of Medicine Ethics Committee: 20130266), and all the participants provided written informed consent.

### Data collection

We collected data on patients’ health insurance, clinical and sociodemographic characteristics, and self-reported outcomes at 3 months after diagnosis.

To assess the realities of private health insurance for patients with advanced lung cancer, we used self-report questions:

1. Status of private health insurance: (1) have both general and cancer-specified private insurance; (2) have general private insurance but no cancer-specified insurance; (3) have no general private insurance but cancer-specified insurance; (4) do not have either general or cancer-specified private insurance

2. Whether their general private insurance includes special coverage for cancer (yes or no)

3. How much their private health insurance helps: (1) fully satisfied; (2) fairly satisfied but insufficient; (3) satisfied to some extent but quite insufficient; (4) hardly satisfied

4. Whether they think that national and/or private health insurance are necessary in general: (1) both national and private health insurance are necessary; (2) national health insurance is necessary but private health insurance is not necessary; (3) national health insurance is not necessary, adequate private health insurance is necessary; (4) neither national nor private health insurance is necessary.

The health-related QOL of patients was measured using the Functional Assessment of Cancer Therapy-Lung scale (FACT-L), which consists of multiple dimensions of QOL: Physical Well-Being, Social Well-Being (SWB), Emotional Well-Being (EWB), Functional Well-Being, and Lung Cancer Symptom Burden ^(16), (17)^. Higher scores indicate better QOL and lower symptom burden.

Mood symptoms of patients were measured using the Hospital Anxiety and Depression Scale (HADS), a 14-item self-report questionnaire that contains 2 subscales measuring HADS-Anxiety and HADS-Depression ^(18), (19)^. Scores for each subscale range from 0 (no distress) to 21 (maximum distress).

### Statical analyses

The data were descriptively analyzed. Comparisons between the 2 groups were performed using the Mann-Whitney U test or Fisher’s exact probability test. For multivariable logistic analysis, we selected age, SWB subscale score, and EWB subscale score as variables based on the preceding univariate comparisons and correlations between the explanatory variables. Specifically, age, but not an Eastern Cooperative Oncology Group Performance Status (ECOG-PS), was included due to the correlation between age and ECOG-PS, the correlations between ECOG-PS and FACT-L scores, and the general fact that age is a major factor when people acquire health insurance. The FACT-L subscales EWB and SWB were included instead of the FACT-L total score. For multivariable linear regression analyses, we selected age, ECOG-PS, clinical stage of cancer, and private health insurance status as variables to examine the impact of private health insurance on QOL while considering the background clinical factors. All p-values were two-sided, and p < 0.05 was considered significant for each test. Data were analyzed using SPSS v. 28.

## Results

### Characteristics of patients

Among the 155 patients who responded to the questionnaire survey 3 months after diagnosis, 147 patients reported whether they had private health insurance. The clinical and sociodemographic characteristics of these 147 patients are shown in Table 1. Most participants in the patient cohort were >60 years old, married, and exhibited an ECOG-PS of 0 to 1. The initial treatment was primarily chemotherapy.

**Table 1.** Clinical and Sociodemographic Characteristics of Patients.

	Patients
	(N = 147)
Age	70 (63-75)
Gender
Male	104 (71.2)
Female	42 (28.8)
Marital status
Married	100 (70.9)
Single/widowed/divorced	41 (29.1)
Employment status
Employed	33 (23.6)
Unemployed	107 (76.4)
Household income (million JPY)	
0-1.99	14 (11.5)
2.00-3.99	52 (42.6)
4.00-5.99	21 (17.2)
6.00-7.99	20 (16.4)
8.00-	15 (12.3)
ECOG performance status	
0	50 (34.7)
1	73 (50.7)
2	16 (11.1)
3	2 (1.4)
4	3 (2.1)
Clinical stage of cancer	
ⅢB	26 (17.8)
Ⅳ	120 (82.2)
Initial treatment
Chemotherapy	121 (88.3)
Radiation	0 (0.0)
Chemoradiation	15 (10.9)
Best supportive care	1 (0.7)

Data are presented as n (%). Age is presented as median [interquartile range]. ECOG: Eastern Cooperative Oncology Group, JPY: Japanese Yen.

**Figure 1.**
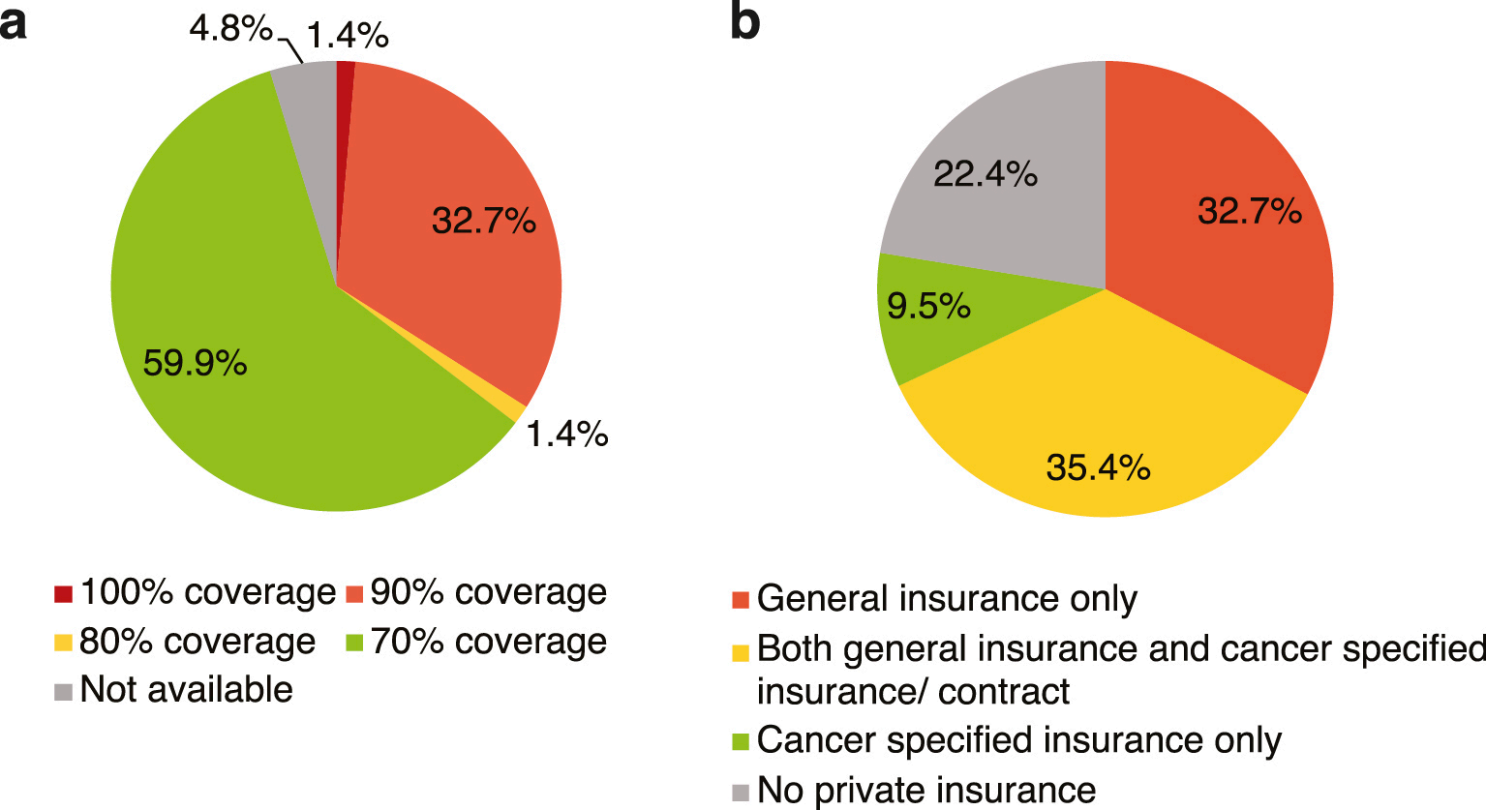
Coverages of the national and private health insurance in patients with advanced lung cancer in Japan. (a) The proportion of the patients with each national health insurance coverage (n = 147). (b) The proportion of the patients with each private health insurance category (n = 147).

The coverage types of the national and private health insurance for the 147 patients are shown in Figure 1. The most frequent category of coverage by national health insurance was 70% coverage (59.8%), followed by 90% (32.7%), 80% (1.4%), and 100% coverage (1.4%) (Figure 1a). Among the 147 patients, 114 patients (77.6%) also had private health insurance: both general insurance and cancer-specific insurance/contract (35.4%), followed by general insurance only (32.7%), and cancer-specific insurance only (9.5%) (Figure 1b).

### Associated factors with having private health insurance

We examined associations between having private health insurance and various variables (Table 2). Patients with private health insurance were significantly younger (p = 0.028) and had better ECOG-PS (p = 0.029). For other background characteristics, there were no significant differences in gender, marital status, employment status, household income, or cancer clinical stage. There was no significant difference in initial treatment between those with and without private health insurance. In addition, there were no significant differences in the presence of financial problems or patients’ satisfaction with medical care between the 2 groups. When we examined whether health-related QOL was associated with having private health insurance, patients with private health insurance showed significantly higher FACT-L total scores (p = 0.017), SWB subscale scores (p = 0.039), and EWB subscale scores (p = 0.011).

**Table 2.** Associations between Having Private Health Insurance and Variables.

	Patients with	Patients without	p-Value
	Private insurance	Private insurance	
	(n = 114)	(n = 33)	
Age	69 (62-75)	74 (64-81)	0.028^a^
Gender			0.829^b^
Male	81 (71.7)	23 (69.7)
Female	32 (28.3)	10 (30.3)
Marital status			0.188^b^
Married	80 (74.1)	20 (60.6)
Single/widowed/divorced	28 (25.9)	13 (39.4)
Employment status			0.103^b^
Employed	29 (26.9)	4 (12.5)
Unemployed	79 (73.2)	28 (87.5)
Household income (million JPY)			0.858^b^
0-1.99	10 (10.5)	4 (14.8)
2.00-3.99	39 (41.1)	13 (48.2)
4.00-5.99	17 (17.9)	4 (14.8)
6.00-7.99	16 (16.8)	4 (14.8)
8.00-	13 (13.7)	2 (7.4)
ECOG performance status			0.029^b^
0	45 (39.8)	5 (16.1)
1	55 (48.7)	18 (58.1)
2	9 (8.0)	7 (22.6)
3	2 (1.8)	0 (0.0)
4	2 (1.8)	1 (3.2)
Clinical stage of cancer			0.451^b^
ⅢB	17 (15.0)	8 (24.2)
Ⅳ	96 (85.0)	25 (75.8)
Initial treatment			0.094^b^
Chemotherapy	97 (87.3)	25 (75.8)
Radiation	0 (0.0)	0 (0.0)
Chemoradiation	14 (12.6)	7 (21.2)
Best supportive care	0 (0.0)	1 (3.0)
Financial problem			0.652^b^
Not at all	61 (56.0)	15 (45.5)
A little	32 (29.4)	12 (36.4)
Quite a bit	12 (11.0)	4 (12.1)
Very much	4 (3.7)	2 (6.1)
Medical satisfaction			0.221^b^
Very satisfied	27 (24.3)	4 (12.1)
Satisfied	57 (51.4)	18 (54.6)
A little satisfied	22 (19.8)	9 (27.3)
A little unsatisfied	5 (4.5)	1 (3.0)
Unsatisfied	0 (0.0)	1 (3.0)
Very unsatisfied	0 (0.0)	0 (0.0)
FACT-L total score	83.5 [72.4-94.1]	75.0 [68.8-87.6]	0.017^a^
Physical Well-Being subscale score	18.0 [14.0-23.0]	18.0 [14.0-22.1]	0.620^a^
Social Well-Being subscale score	18.7 [15.2-22.2]	15.8 [12.8-21.0]	0.039^a^
Emotional Well-Being subscale score	17.0 [12.5-21.0]	14.0 [11.5-17.5]	0.011^a^
Functional Well-Being subscale score	16.7 [12.0-20.0]	14.5 [9.5-18.5]	0.104^a^
Lung Cancer subscale score	15.0 [13.0-17.0]	14.0 [13.0-15.0]	0.448^a^
HADS-Anxiety score	4 [2-7]	5 [3-7]	0.164^a^
HADS-Depression score	6 [2-9]	8 [4-11]	0.068^a^

Data are presented as n (%), and median [interquartile range]. ^a^Mann-Whitney U test. ^b^Fisher’s exact probability test. ECOG: Eastern Cooperative Oncology Group; FACT-L: Functional Assessment of Cancer Therapy-Lung; HADS: Hospital Anxiety and Depression Scale; JPY: Japanese Yen.

In multivariable analysis, younger age (odds ratio [OR], 0.944; 95% clinical interval [CI], 0.901-0.989; p = 0.016), higher SWB subscale score (OR, 1.082; 95% CI, 1.001-1.168; p = 0.047), and higher EWB subscale score (OR, 1.094; 95% CI, 1.007-1.190; p = 0.035) were significantly associated with having private health insurance (Table 3).

**Table 3.** Multivariable Logistic Analysis of Associated Factors with Having Private Health Insurance.

	OR	95% CI	p-Value
Age	0.944	0.901-0.989	0.016
Social Well-Being Subscale score	1.082	1.001-1.168	0.047
Emotional Well-Being Subscale score	1.094	1.007-1.190	0.035

CI: confidence interval; OR: odds ratio.

### Impact of private health insurance on QOL

Next, we investigated the impact of having private health insurance on QOL, as well as the impact of major clinical factors, such as age, ECOG-PS, and clinical stage of cancer, all of which could affect patients’ QOL (Table 4). Notably, while ECOG-PS was a significant predictor of FACT-L total score and EWB subscale score, having private health insurance (but not ECOG-PS) was an independent predictor of SWB subscale score (p = 0.044).

**Table 4.** Linear Regression Analysis of Associated Factors with QOL Scores.

	Univariable		Multivariable	
	B (95% CI)	*p*-value	B (95% CI)	p-Value
FACT-L total score			
Age	0.009 (−0.261 to 0.278)	0.950	0.150 (−0.111 to 0.410)	0.258
ECOG performance status	−8.729 (−11.84 to -5.615)	<0.001	−8.828 (−12.05 to −5.605)	<0.001
Clinical stage of cancer	4.224 (−3.062 to 11.35)	0.254	5.652 (−1.024 to 12.33)	0.096
Private health insurance	7.596 (0.961-14.23)	0.025	3.387 (−3.163 to 9.936)	0.308
SWB subscale score
Age	0.006 (−0.083 to 0.095)	0.893	0.056 (−0.038 to 0.150)	0.240
ECOG performance status	−0.946 (−2.082 to 0.189)	0.102	−0.827 (−1.981 to 0.326)	0.158
Clinical stage of cancer	2.047 (−0.355 to 4.449)	0.094	2.027 (−0.396 to 4.450)	0.100
Private health insurance	2.693 (0.479-4.907)	0.017	2.428 (0.630-4.793)	0.044
EWB subscale score
Age	0.015 (−0.065-0.096)	0.706	0.055 (−0.026 to 0.137)	0.183
ECOG performance status	−1.950 (−2.925 to −0.976)	<0.001	−2.020 (−3.024 to −1.016)	<0.001
Clinical stage of cancer	1.370 (−0.812 to 3.552)	0.216	1.784 (−0.309 to 3.878)	0.094
Private health insurance	2.273 (0.288-4.259)	0.025	1.316 (−0.733 to 3.365)	0.206

B: beta regression coefficient; CI: confidence interval; ECOG: Eastern Cooperative Oncology Group; EWB: Emotional Well-Being; FACT-L: Functional Assessment of Cancer Therapy-Lung; SWB: Social Well-Being.

### Patients’ satisfaction with private health insurance and perception of the necessity for private health insurance

**Figure 2.**
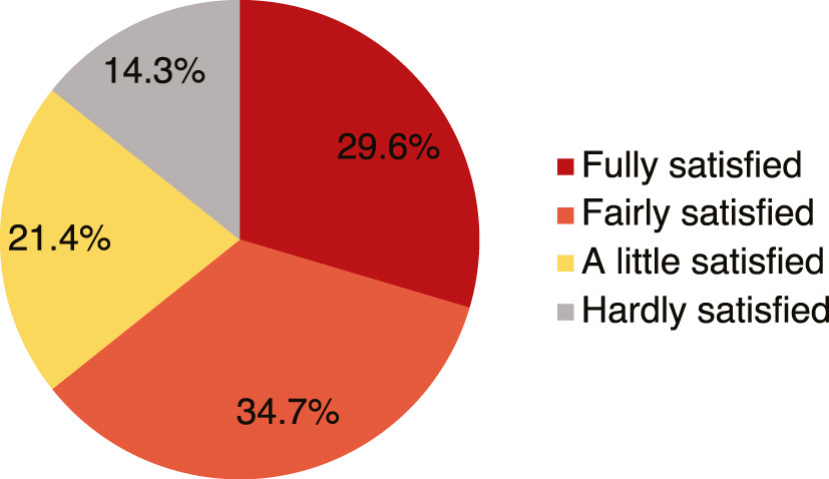
Patient satisfaction levels with private health insurance (n = 98).

Patients’ satisfaction levels with private health insurance are shown in Figure 2. Of the 98 patients who answered the satisfaction survey, 29 patients (29.6%) were “fully satisfied,” 34 (34.7%) were “fairly satisfied but insufficient,” 21 (21.4%) were “a little satisfied but not enough,” and 14 (14.3%) were “hardly satisfied.”

**Figure 3.**
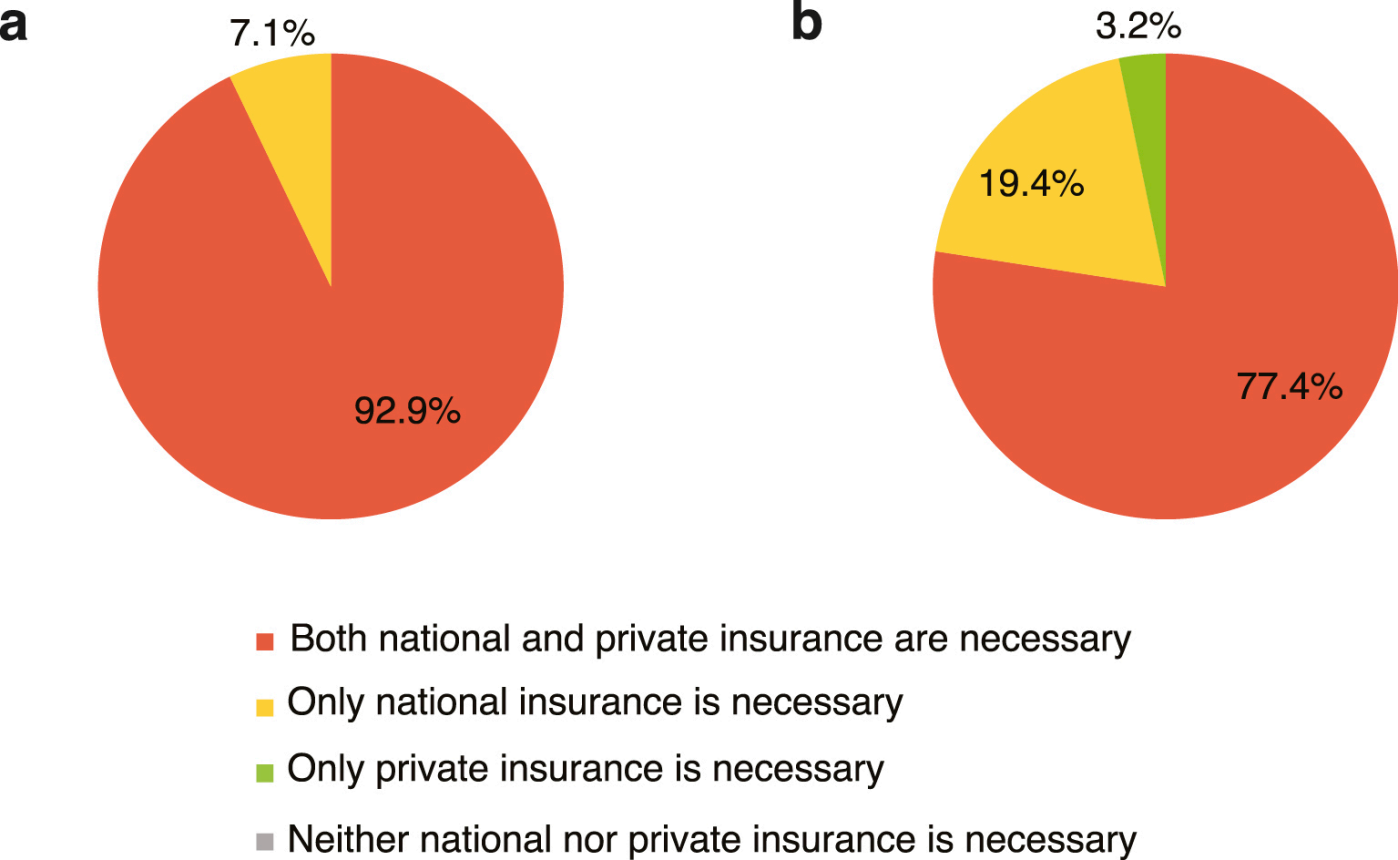
Perception of the necessity for health insurance in patients diagnosed with advanced lung cancer. (a, b) Answers to the question of whether national and private health insurance is necessary for (a) patients with private health insurance (n = 112) and (b) without private health insurance (n = 31).

Responses to the question of whether national and private health insurance are necessary are shown in Figure 3. A total of 143 patients (112 with private health insurance and 31 without it) responded, with most patients (99.3%) agreeing that national health insurance is necessary. While 7.1% of those with private health insurance felt private health insurance was not necessary, 19.4% of those without private health insurance felt the same.

## Discussion

In this multicenter study, we investigated the factors associated with having private health insurance and found that patients with private health insurance had better QOL. To the best of our knowledge, this is the first study to comprehensively survey health insurance and demonstrate the association between having private health insurance and QOL in a Japanese clinical setting, providing insight into the structure of health insurance in Japan.

It is noteworthy that the FACT-L total scores, SWB, and EWB subscale scores―measures of QOL―were significantly higher for patients with private health insurance. In addition, multivariable analysis revealed that those with private health insurance had higher SWB subscale scores. It is possible that private health insurance mitigates financial toxicity, resulting in improved social and emotional QOL. Conversely, we can speculate that patients with good social and emotional backgrounds are more likely to have private health insurance. Previous studies have reported that an increased economic burden is associated with lower physical, mental, and social QOL ^(20), (21), (22)^. However, physical and functional QOL were not related to private health insurance coverage in this study, which may be due to the low percentage of patients in Japan who refrain from seeking medical care due to economic problems. Additionally, treatment options do not differ significantly depending on patients’ economic situations. It has been reported that only 2.9% of those aged 20-65 and 1.1% of those aged ≥65 did not visit a healthcare provider for financial reasons in the previous year ^(23)^. In this study, no significant differences in initial treatment details, patient satisfaction, or the presence of financial problems were found between patients with private health insurance and those without. Our findings suggest that the national health insurance system in Japan provides at least the minimally required financial support to every patient with advanced lung cancer.

In terms of patient background, patients with private health insurance were younger and had better ECOG-PS scores. A previous study in South Korea found that patients with private health insurance tended to be younger, which is consistent with our study ^(24)^. This may be because the role of insurance is to provide for future risks. Although previous reports have indicated that higher income is associated with private insurance coverage ^(25), (26)^, no significant correlation between household income and having private insurance was found in this study. This could be because many patients with lung cancer are elderly, retired, and have less income, which does not necessarily mean they have less financial leeway without information on their financial assets.

Among patients with private health insurance, 64.3% were at least fairly satisfied with their insurance coverage. Since the questionnaire did not inquire about the reasons for satisfaction or dissatisfaction, the details are unavailable. It is possible that their private health insurance does not sufficiently help patients with advanced cancer. In fact, patients with private health insurance reported financial problems similar to those without private health insurance.

Notably, a substantial proportion of patients, especially those without private health insurance, reported that private health insurance is not necessary. Since patients with low income and elderly patients are financially well supported by the Japanese medical care system, Japanese patients may not perceive private insurance as a necessity.

Our study has a few limitations. First, our survey method for assessing health insurance using the questionnaire has not been validated, and we did not inquire about the details of patients’ private health insurance. Second, the range of our survey items might not have been sufficient. For example, one might speculate on the relationship between financial status and having private health insurance; however, we did not ask about all of the patients’ assets. Although we collected information on their income and employment status, these were not associated with having private health insurance, probably because most patients were already retired. Third, this study is cross-sectional, and therefore, causal relationships between having private health insurance and other variables cannot be estimated.

In conclusion, we found that patients with private health insurance had better QOL, while there were no significant differences in the presence of financial problems or treatment details between those with and without private health insurance. Private health insurance in Japan plays a supplementary rather than indispensable role in receiving medical care; nevertheless, most patients with advanced lung cancer recognize the necessity of private health insurance.

## Article Information

### Conflicts of Interest

Takashi Sato reports honoraria from Chugai Pharma, Bristol-Myers Squibb, AstraZeneca, Ono Pharmaceutical, Nippon Kayaku, Eli Lilly, Boehringer Ingelheim, Daiichi-Sankyo, Takeda Pharmaceutical and Merck Sharp & Dohme (MSD). Daisuke Arai reports Chugai Pharma, AstraZeneca, Ono Pharmaceutical, Nippon Kayaku, Taiho Pharmaceutical, and MSD. Keiko Ohgino reports honoraria from Chugai Pharma, Bristol-Myers Squibb, AstraZeneca, Ono Pharmaceutical, Taiho Pharmaceutical, Takeda Pharmaceutical, Boehringer Ingelheim, and Merck. Kota Ishioka reports honoraria from AstraZeneca and Chugai Pharma. Hideki Terai reports honoraria from AstraZeneca, Chugai Pharma, Ono Pharmaceutical, Eli Lilly, Takeda Pharmaceutical, Taiho Pharmaceutical, Daiichi-Sankyo, and Bristol-Myers Squibb. Shinnosuke Ikemura reports honoraria from AstraZeneca, Chugai Pharma, and Takeda Pharmaceutical. Hiroyuki Yasuda reports honoraria from AstraZeneca, Ono Pharmaceutical, Bristol-Myers Squibb, Taiho Pharmaceutical, Chugai Pharma, MSD, Takeda Pharmaceutical, Eli Lilly, Daiichi-Sankyo and grants/contracts from Boehringer Ingelheim, Eli Lilly, Novartis and Daiichi-Sankyo, Ichiro Nakachi reports honoraria from AstraZeneca, Fisher Scientific, Chugai Pharma and Takeda Pharmaceutical. Ichiro Kawada reports honoraria from Chugai Pharma, Daiichi-Sankyo, Eli Lilly, Nippon Kayaku, and Bayer. Takashi Inoue reports honoraria from Boehringer Ingelheim, Kyorin Pharmaceutical, Takeda Pharmaceutical, Taiho Pharmaceutical, Chugai Pharma, Sanofi, Insmed, AstraZeneca, Nippon Kayaku, Shionogi, Kyowa Kirin, and Bristol-Myers Squibb. Yoshitaka Oyamada reports honoraria from Chugai Pharma, Ono Pharmaceutical, AstraZeneca, Novartis, MSD, Nippon Kayaku, Sanofi, and GlaxoSmithKline, and grants/contracts from Chugai Pharma, AstraZeneca, Novartis, and Pfizer. Takeshi Terashima reports honoraria from Chugai Pharma, Eli Lilly, Boehringer Ingelheim, MSD, AstraZeneca, Taiho Pharmaceutical, Kyowa Kirin, Novartis, Sanofi, and GlaxoSmithKline, and grants/contracts from Boehringer Ingelheim, Chugai Pharma, Taiho Pharmaceutical and Boehringer Ingelheim. Koichi Sayama reports honoraria from Boehringer Ingelheim. Daisuke Fujisawa reports honoraria from Eisai, Yoshitomi, Pfizer, MSD, Daiichi-Sankyo, and Shionogi, and grants/contracts from Eisai, the Swiss Chamber of Commerce. Mari Takeuchi reports honoraria from Kyowa Kirin, Daiichi-Sankyo and Shionogi, and grants/contracts from Kyowa Kirin, Daiichi-Sankyo, Shionogi, and Maruishi Pharmaceutical. Koichi Fukunaga reports honoraria from Boehringer Ingelheim, Novartis, Sanofi, GlaxoSmithKline, AstraZeneca, and grants/contracts from Boehringer Ingelheim, Ono Pharmaceutical, Chugai Pharma, Taiho Pharmaceutical. Katsuhiko Naoki reports honoraria from Bristol-Myers Squibb, AstraZeneca, Chugai Pharma, Pfizer, Boehringer Ingelheim, Daiichi-Sankyo, Eli Lilly, MSD, Taiho Pharmaceutical, Novartis, grants/contracts from Boehringer Ingelheim. Kenzo Soejima reports honoraria from AstraZeneca, Chugai Pharma, Ono Pharmaceutical, Eli Lilly, Takeda Pharmaceutical, Taiho Pharmaceutical, Novartis, MSD, Bristol-Myers Squibb, Pfizer, Boehringer Ingelheim, Sanofi, grants/contracts from Taiho Pharmaceutical and AstraZeneca.

### Acknowledgement

The authors thank all the participants and collaborators involved in this study. The authors also thank Ms. Chiyomi Uemura and Ms. Kimiko Ito for assisting with data collection, and Editage (www.editage.com) for English language editing.

### Author Contributions

Fumimaro Ito, Takashi Sato, Kohei Horiuchi, Daisuke Arai, Keiko Ohgino, Kota Ishioka, Daisuke Fujisawa, Mari Takeuchi, Katsuhiko Naoki, and Kenzo Soejima conceived and designed the study, Takashi Sato, Daisuke Arai, Keiko Ohgino, Kota Ishioka, Hideki Terai, Shinnosuke Ikemura, Hiroyuki Yasuda, Ichiro Nakachi, Ichiro Kawada, Takashi Inoue, Yoshitaka Oyamada, Takeshi Terashima, Koichi Sayama, Koichi Fukunaga, Katsuhiko Naoki, and Kenzo Soejima collected the data. Fumimaro Ito, Takashi Sato, and Kohei Horiuchi analyzed the data. All authors contributed to the interpretation of the results and critical revision of the manuscript for valuable intellectual content. Takashi Sato, Daisuke Arai, and Kenzo Soejima supervised the conduct of the whole study. Fumimaro Ito and Takashi Sato drafted the manuscript. All authors had access to the data and have read and approved the final manuscript.

### Approval by Institutional Review Board (IRB)

This study was approved by the institutional review board of all participating hospitals (Keio University School of Medicine Ethics Committee: 20130266), and all the participants submitted a written informed consent.

## References

[1] Elkinton JR. Medicine and the quality of life. Ann Intern Med. 1966;64(3):711-4. 5324639 10.7326/0003-4819-64-3-711

[2] Sato T, Fujisawa D, Arai D, et al. Trends of concerns from diagnosis in patients with advanced lung cancer and their family caregivers: a 2-year longitudinal study. Palliat Med. 2021;35(5):943-51. 33761790 10.1177/02692163211001721 PMC8114458

[3] Smith GL, Lopez-Olivo MA, Advani PG, et al. Financial burdens of cancer treatment: a systematic review of risk factors and outcomes. J Natl Compr Canc Netw. 2019;17(10):1184-92. 31590147 10.6004/jnccn.2019.7305 PMC7370695

[4] Zafar SY. Financial toxicity of cancer care: it’s time to intervene. J Natl Cancer Inst. 2016;108(5):djv370. 26657334 10.1093/jnci/djv370

[5] Davidoff AJ, Erten M, Shaffer T, et al. Out-of-pocket health care expenditure burden for Medicare beneficiaries with cancer. Cancer. 2013;119(6):1257-65. 23225522 10.1002/cncr.27848

[6] Ikegami N, Yoo BK, Hashimoto H, et al. Japanese universal health coverage: evolution, achievements, and challenges. Lancet. 2011;378(9796):1106-15. 21885107 10.1016/S0140-6736(11)60828-3

[7] Health at a glance 2021 [Internet]. OECD. OECD Indicators; 2021. [cited 2023 Jan 8]. Available from: https://www.oecd-ilibrary.org/social-issues-migration-health/health-at-a-glance-2021_ae3016b9-en

[8] Matsuda S. Health policy in Japan - current situation and future challenges. JMA J. 2019;2(1):1-10. 33681508 10.31662/jmaj.2018-0016 PMC7930804

[9] Thomas R. Health insurance systems: an international comparison. Amsterdam: Elsevier; 2021. 366 p.

[10] Matsuda R. International health care system profiles [Internet]. The Commonwealth Fund; 2020. [cited 2023 Jan 8]. Available from: https://www.commonwealthfund.org/international-health-policy-center/countries/japan

[11] Survey on livelihood security [Internet]. Japan Institute of Life Insurance. 2022 [cited 2023 Jan 8]. Available from: https://www.jili.or.jp/files/research/chousa/pdf/r4/2022hosho.pdf

[12] Sato T, Soejima K, Fujisawa D, et al. Prognostic understanding at diagnosis and associated factors in patients with advanced lung cancer and their caregivers. Oncologist. 2018;23(10):1218-29. 30120158 10.1634/theoncologist.2017-0329 PMC6263137

[13] Kameyama N, Sato T, Arai D, et al. Most important things and associated factors with prioritizing daily life in patients with advanced lung cancer. JCO Oncol Pract. 2022;18(12):e1977-86. 36346964 10.1200/OP.22.00124

[14] Arai D, Sato T, Nakachi I, et al. Longitudinal assessment of prognostic understanding in patients with advanced lung cancer and its association with their psychological distress. Oncologist. 2021;26(12):e2265-73. 34510654 10.1002/onco.13973 PMC8649026

[15] Goldstraw P, Crowley J, Chansky K, et al. The IASLC Lung Cancer Staging Project: proposals for the revision of the TNM stage groupings in the forthcoming (seventh) edition of the TNM Classification of malignant tumours. J Thorac Oncol. 2007;2(8):706-14. 17762336 10.1097/JTO.0b013e31812f3c1a

[16] Cella DF, Bonomi AE, Lloyd SR, et al. Reliability and validity of the Functional Assessment of Cancer Therapy-Lung (FACT-L) quality of life instrument. Lung Cancer. 1995;12(3):199-220. 7655830 10.1016/0169-5002(95)00450-f

[17] Saitoh E, Yokomizo Y, Chang CH, et al. Cross-cultural validation of the Japanese version of the lung cancer subscale on the functional assessment of cancer therapy-lung. J Nippon Med Sch. 2007;74(6):402-8. 18084133 10.1272/jnms.74.402

[18] Zigmond AS, Snaith RP. The hospital anxiety and depression scale. Acta Psychiatr Scand. 1983;67(6):361-70. 6880820 10.1111/j.1600-0447.1983.tb09716.x

[19] Kitamura T. The hospital anxiety and depression scale. Arch Psychiatr Diagn Clin Eval. 1993;4:2.

[20] Fenn KM, Evans SB, McCorkle R, et al. Impact of financial burden of cancer on survivors’ quality of life. J Oncol Pract. 2014;10(5):332-8. 24865220 10.1200/JOP.2013.001322

[21] Kale HP, Carroll NV. Self-reported financial burden of cancer care and its effect on physical and mental health-related quality of life among US cancer survivors. Cancer. 2016;122(8):283-9. 26991528 10.1002/cncr.29808

[22] Park J, Look KA. Relationship between objective financial burden and the health-related quality of life and mental health of patients with cancer. J Oncol Pract. 2018;14(2):e113-21. 29381411 10.1200/JOP.2017.027136

[23] Sakamoto H, Rahman M, Nomura S, et al. Japan health system review. Health Syst Transit. Regional Office for South-East Asia. World Health Organization; 2018 [cited 2023 Jan 8]. Available from: https://apps.who.int/iris/handle/10665/259941

[24] Shin DW, Jung KT, Kim S, et al. Impact of supplementary private health insurance on stomach cancer care in Korea: a cross-sectional study. BMC Health Serv Res. 2009;9:133. 19643032 10.1186/1472-6963-9-133 PMC2726135

[25] National survey on life insurance [Internet]. Japan Institute of Life Insurance; 2021 [cited 2023 Jan 8]. Available from: https://www.jili.or.jp/research/report/8361.html

[26] Lim JH, Kim SG, Lee EM, et al. The determinants of purchasing private health insurance in Korean cancer patients. J Prev Med Public Health. 2007;40(2):150-4. 17426427 10.3961/jpmph.2007.40.2.150

